# Bioethanol from Lignocellulosic Biomass: Current Findings Determine Research Priorities

**DOI:** 10.1155/2014/298153

**Published:** 2014-12-31

**Authors:** Qian Kang, Lise Appels, Tianwei Tan, Raf Dewil

**Affiliations:** ^1^Key Lab of Bioprocess, College of Life Science and Technology, Beijing University of Chemical Technology, Beijing 100029, China; ^2^Department of Chemical Engineering, Process and Environmental Technology Lab, KU Leuven, 2860 Sint-Katelijne-Waver, Belgium

## Abstract

“Second generation” bioethanol, with lignocellulose material as feedstock, is a promising alternative for first generation bioethanol. This paper provides an overview of the current status and reveals the bottlenecks that hamper its implementation. The current literature specifies a conversion of biomass to bioethanol of 30 to ~50% only. Novel processes increase the conversion yield to about 92% of the theoretical yield. New combined processes reduce both the number of operational steps and the production of inhibitors. Recent advances in genetically engineered microorganisms are promising for higher alcohol tolerance and conversion efficiency. By combining advanced systems and by intensive additional research to eliminate current bottlenecks, second generation bioethanol could surpass the traditional first generation processes.

## 1. Introduction

### 1.1. Bioethanol as Sustainable Fuel

With the global increasing demand for energy, energy shortage will be a global problem. Bioethanol is considered as an important renewable fuel to partly replace fossil-derived fuels. The world production of bioethanol increased from 50 million m^3^ in 2007 to over 100 million m^3^ in 2012 [1]: Brazil and the United States represent approximately 80% of the world supply, mostly using corn or sugarcane. In developing economies, food-related feedstock is preferably replaced by nonfood raw materials, such as sweet sorghum or cassava. The use of common biomass could significantly increase the bioethanol production, and lignocellulose-based bioethanol is therefore the topic of the present review paper. The current technological development and bottlenecks define the short- and medium-term research priorities.

Industrial ethanol is mainly produced petrochemically through the acid-catalyzed hydration of ethylene. Ethanol for use in alcoholic beverages, and the vast majority of ethanol for use as biofuel, is produced by fermentation where certain species of yeast (e.g.,* Saccharomyces cerevisiae*) or bacteria (e.g.,* Zymomonas mobilis*) metabolize sugars in oxygen-lean conditions to produce ethanol and carbon dioxide.

The main reasons for the enhanced development of bioethanol are its use as a favourable and near carbon-neutral renewable fuel, thus reducing CO_2_ emissions and associated climate change; its use as octane enhancer in unleaded gasoline; and its use as oxygenated fuel-mix for a cleaner combustion of gasoline, hence reducing tailpipe pollutant emissions and improving the ambient air quality. The largest single use of ethanol is as engine fuel and fuel additive, with common types of available fuel-mixes listed in Table 1.

Ethanol has appropriate properties for spark ignition IC engines. Its octane numbers, motor octane number (MON) and research octane number (RON), are 90 and 109, respectively, on average 99 compared to 91 for regular gasoline. Due to its low cetane number, ethanol does not burn efficiently by compression ignition and is moreover not easily miscible with diesel fuel. To improve the use of ethanol in compression-ignition (CI) engine vehicles, measures can be taken, such as the addition of an emulsifier in order to increase the ethanol-diesel miscibility; the addition of ethylhexyl nitrate or diterbutyl peroxide to enhance the cetane number; the use of a dual fuel operation in which ethanol and diesel are introduced separately into the cylinder; or the modification of diesel engines in order to adapt their characteristics of autoignition [2].

### 1.2. Bioethanol from Different Feedstocks

Fermentation of sugar-based raw materials is referred to as “first generation” bioethanol, whereas the use of lignocellulose raw materials is commonly called “second generation” bioethanol. The “third generation” of algal bioethanol is at an early stage of investigation. The first generation processes were discussed in detail by Kang et al. [1]. Since the present paper deals with second generation bioethanol, relevant and recent (>2010) literature is summarized in Table 2. The 2nd generation bioethanol processes will use cellulose-released sugars, despite the cost of the required enzymes to hydrolyse cellulose. Development of this technology could deal with a number of cellulose-containing agricultural byproducts, such as straw, wood trimmings, sawdust, bamboo, and others.

The specificities of using lignocellulosic raw materials will be dealt with in Section 1.3. Whether first, second, or third generation feedstock is used, fermentation produces an alcohol-lean broth only, as such unusable in industrial and fuel applications. The ethanol must hence be purified. Fractional distillation can concentrate ethanol to 95.6 vol% (89.5 mol%), corresponding to the azeotropic composition with a boiling point of 78.2°C. Further ethanol enrichment by common distillation is impossible, but different alternatives have been investigated, as reported by Kang et al. [3].

### 1.3. Lignocellulosic Biomass

#### 1.3.1. Sources

Available biomass can be categorized into primary sources, produced as either crop or key product, for example, sugar cane, short rotation energy plantations; secondary sources, as residues from the production processes, for example, bagasse, rice husks, and straw; and tertiary sources, as residues produced during and after application end, for example, the organic fraction of municipal solid waste (MSW), sewage treatment sludge, wood trimmings, and so forth [4]. In general the final availability of organic wastes and residues may fluctuate and is affected by market growth, although climate and other factors have influences, especially when considering the primary sources. The energy potential of biomass residues and organic wastes depends on the yield, the total land area available, and the type of production.

The organic fraction of municipal solid waste (MSW) is an inexpensive source of biomass and covers domestic and industrial waste collected in a specific area. The overall potential of the organic fraction of municipal waste and its waste wood fraction is strongly reliant on economic growth, consumption, and the use of biomaterials. It is estimated at between 5 and 50 EJ/year [4]. The unit EJ (eta-joule) is equal to 10^18^ J.

Agroindustrial biomass residues are byproducts of agriculture or its related industry, including cotton stalks, wheat and rice straw, coconut shells, maize cobs, jute sticks, and rice husks [5]. The agricultural residues are produced decentralised and have a low density. Due to the high transportation cost, it is expensive to apply agricultural residues as the main fuel in power stations. The potential of agriculture residues varies from 15 up to 70 EJ/year [4], as a function of regional production, harvesting processing factors, and recoverability factors. Within the agroindustrial residues, dried manure is considered as a tertiary source. A total worldwide estimate is difficult to make, and given as 5 to 55 EJ/year, with the lower estimate due to the current use as fertiliser, while the higher estimate considers the total technical potential [5].

Forestry residues include biomass, not harvested or removed from sorting regions in commercial hardwood and softwood production, through forest management operations such as precommercial thinning and removal of dead and dying trees. Forestry waste includes wood chips, sawdust, and bark. It can provide 65% of the biomass energy potential [6, 7]. The extraction costs and the required transportation to centralized processing plants make forest fuels expensive. Several studies have focused on their use for energy production at a district level, applying appropriate designs of decentralized smaller plants, for example, Malinen et al. [8] and Demirbaş [9]. The energy potential of the world's forests is again difficult to estimate. The possible contribution by 2050 is estimated at 98 EJ/year of excess natural forest growth and at 32–52 EJ/year of processing residues. Although these lignocellulosic biomass resources represent a significant energy value, ~150 EJ/year, only part of this resource can be used as feedstock for bioethanol, for reasons given below.

#### 1.3.2. Composition

Lignocellulose, the principal component of the plant cell walls, is mainly composed of cellulose (40–60% of the total dry weight), hemicellulose (20–40%), and lignin (10–25%). Cellulose consists of long chains of *β*-glucose monomers gathered into microfibril bundles. The hemicelluloses, mostly xyloglucans or xylans, are linked to the microfibrils by hydrogen bonds. Lignins are phenolic compounds which are formed by polymerisation of three types of monomers (p-coumaryl, coniferyl, and synapyl alcohols). Lignin adds compressive strength and stiffness to the cell wall [10]. Once the lignocellulosic biomass is pretreated and hydrolysed, the released sugars can be fermented and the downstream process is similar to that of first generation feedstock [1]. Potential lignocellulosic feedstocks and their composition are summarized in Table 3.

High lignin and/or high ash concentrations are unfavorable for bioethanol production. Softwood especially can hence be excluded. The extensive hydrogen linkages among cellulose molecules lead to a crystalline and strong matrix structure [11]. Although starches require temperatures of only 60–70°C to be converted from crystalline to amorphous texture, cellulose requires 320°C as well as high pressures (up to 25 MPa) to transform the rigid crystalline structure into an amorphous structure in water [12]. Cotton, flax, and chemical pulps represent the purest sources of cellulose, while soft and hardwoods contain less than 50% of cellulose, as shown in Table 4.

Hemicellulose is an amorphous structure formed of different heteropolymers including hexoses (D-glucose, D-galactose, and D-mannose) as well as pentose (D-xylose and L-arabinose). It may contain sugar acids (uronic acids) [13]. Its backbone chain is primarily composed of xylan linkages including *α*-xylose (~90%) and L-arabinose (~10%) [14]. The degree of branching and the xylan composition vary with the nature and the source of raw materials. To be totally hydrolysed into free monomers, hemicellulose requires a wide range of enzymes in view of the diversity of its sugars.

Lignin is an aromatic and rigid biopolymer, covalently bonded to hemicellulosic xylans and responsible for the rigidity and high level of compactness of the plant cell wall [15]. Lignin is composed of monomers of phenyl propionic alcohol, that is, coumaryl, coniferyl, and sinapyl alcohol. The lignin fraction in biomass sources varies considerably, as illustrated in Table 4. Lignin components are gaining importance because of their dilution effect on the processes of hydrolysis and fermentation [16, 17]. The phenolic groups, formed from the degradation of lignin, substantially deactivate cellulolytic enzymes and hence hamper enzymatic hydrolysis. Chen et al. [17] however demonstrated that lignin modification via genetic engineering could considerably reduce lignin formation and improve ethanol yield. This could however be problematic as lignin components serve as the major plant defence system to pathogens and insects and its modification could disrupt the plants' natural protection [18]. Retaining the lignin could moreover benefit the energy-economy of the process, since once recovered, it can be applied in a combined heat and power unit (CHP), thus being a potential energy self-sustaining source of the process. Biomass feedstock with a high lignin content is not readily applicable as raw material for the bioethanol fermentation. This certainly eliminates most of the soft woods.

The different composition of biomass feedstock (dry) is also reflected in its elemental composition. The exact composition is largely dependent on the biomass sources. C, H, and O are the key components of biomass and largely determine their calorific value. Some typical values of the C-, H-, and O-content, as well as other essential data are summarized in Table 4, as a result of an extensive literature survey [9, 19, 20] and own analyses.

The ash content of biomass sources is generally low, as illustrated in Table 5. Its composition should, however, be taken into consideration, since most of the ash will concentrate in the lignin residue, thus possibly hampering further energy generation by fouling or sintering. Biomass contains a significant content of K, Cl, and Si as well as lower concentrations of Ca, Mg, Al, Fe, and Na. The ash content and its chemical composition are a strong function of the biomass species. The elements in the ash are O, Ca, K, Si, Mg, Al, S, Fe, P, Cl, Na, Mn, and Ti [21]. An extensive literature survey was provided by several authors [19, 22, 23] and some examples are summarized in Table 5.

## 2. Processing of Biomass to Ethanol

### 2.1. Generalities

**Figure 1:**
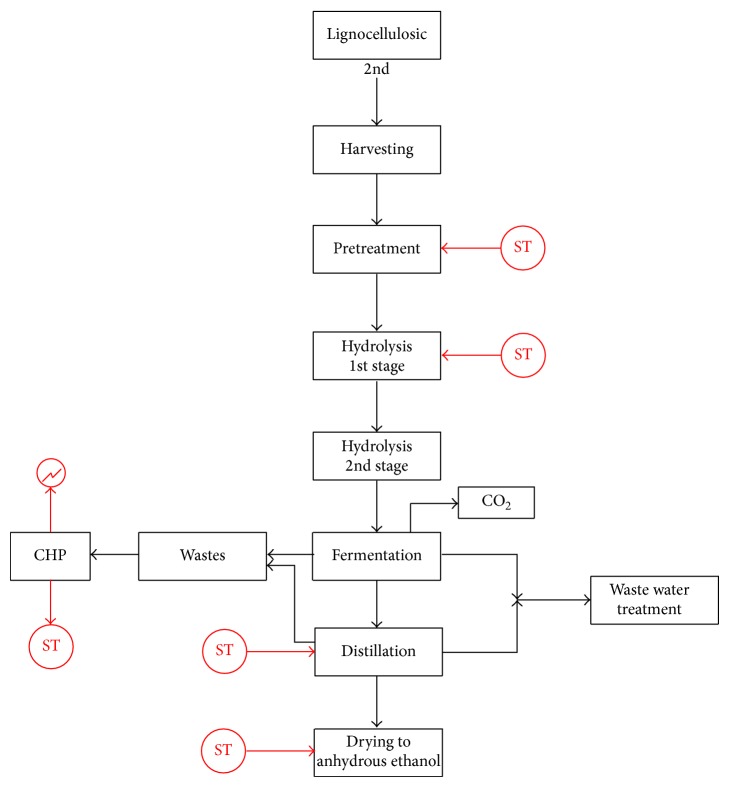
Second generation biomass-to-ethanol production (ST: steam addition).

Once the feedstock is delivered to the ethanol plant, it needs to be carefully stored and conditioned to prevent early fermentation and bacterial contamination. Through pretreatment, simple sugars are made available in proportions depending on the type of biomass used and the pretreatment process. The main steps are summarized in Figure 1, providing a general production flow sheet.

### 2.2. Pretreatment and First Stage Hydrolysis

Pretreatment involves delignification of the feedstock [24] in order to make cellulose more accessible in the hydrolysis step, using physical, physicochemical, chemical, and biological treatment (Table 6). Carbonic acid and alkaline extraction have the best performance. However, the most common methods are steam explosion and dilute acid prehydrolysis, which are followed by enzymatic hydrolysis. Sulphuric acid or carbon dioxide is often added in order to reduce the production of inhibitors and improve the solubilisation of hemicellulose [15]. Steam explosion has a few limitations since the lignin-carbohydrate matrix is not completely broken down; degradation products are generated that reduce the efficiency of the hydrolysis and fermentation steps; and a portion of the xylan fraction is destroyed.

The use of dilute sulphuric acid (0.5–1%; 433–463 K for 10 minutes) has the preference of the US National Renewable Energy Laboratory [25]: hemicellulose is largely hydrolysed releasing different simple sugars (xylose, arabinose, mannose, and galactose), but also other compounds of the cellulosic matrix can however inhibit the enzymatic hydrolysis and fermentation. Part of the acetic acid, much of the sulphuric acid and other inhibitors produced during the degradation of the materials need to be removed, and neutralisation is performed before fermentation. Pretreatment is a costly separation, accounting for approximately 33% of the total cost [26]: the economy needs to be improved, and the release of microbial and chemical contamination that possibly reduces the overall yield needs further attention.

### 2.3. Second Stage Hydrolysis

In the second stage hydrolysis, the released cellulose of the biomass is converted into glucose, which is again catalysed by dilute acid, concentrated acid, or preferably by cellulase enzymes, either produced in a separate reactor or bought externally from industrial suppliers [27–30].

The conversion of cellulose and hemicellulose can be expressed by the reaction of glucan (for hexoses) and xylan (for pentose) with water:

(1) $$\begin{array}{c} {\left({\text{C}}_{6}{\text{H}}_{10}{\text{O}}_{5}\right)}_{n}+n{\text{H}}_{2}\text{O}⟶n{\text{C}}_{6}{\text{H}}_{12}{\text{O}}_{6} \end{array}$$

(2) $$\begin{array}{c} {\left({\text{C}}_{5}{\text{H}}_{8}{\text{O}}_{4}\right)}_{n}+n{\text{H}}_{2}\text{O}⟶n{\text{C}}_{5}{\text{H}}_{10}{\text{O}}_{5} \end{array}$$

The maximum theoretical yield of hexoses and pentoses is 1.136 kg and 1.111 kg per kg of glucan and xylan, respectively.

To overcome inhibition by hydrolyte components, membrane techniques have been investigated [3, 31]. Chandel et al. [32] investigated the strategies that have been adopted to detoxify lignocellulosic hydrolysates and their effects on the chemical composition of the hydrolysates to improve the fermentability of lignocellulosics. Hydrolysis of myco-LB (LB after fungal pretreatment) has been recognized as a promising approach to avoid fermentation inhibitors and improve total sugar recovery. Genetic manipulation could modify the metabolic routes to produce bioethanol or other value-added compounds in an efficient manner. Further research is certainly required, as described in Section 3.

### 2.4. Fermentation

Contrarily to the conversion of disaccharides and starch to ethanol, which are mature technologies, modern lignocellulose-to-ethanol processes are at pilot and demonstration stage: NREL (USA) [25], Iogen Corporation (Canada) [33], and ETEK (Sweden) [34] have built pilot plants capable of producing a few hundred thousand litres of ethanol per year.

Fermentation is the biological process to convert the hexoses and pentoses into ethanol by a variety of microorganisms, such as bacteria, yeast, or fungi. The conversion reaction for hexoses (C6) and pentoses (C5) is as follows:

(3) $$\begin{array}{c} {\text{C}}_{6}{\text{H}}_{12}{\text{O}}_{6}⟶2{\text{C}}_{2}{\text{H}}_{5}\text{OH}+2\text{C}{\text{O}}_{2} \end{array}$$

(4) $$\begin{array}{c} 3{\text{C}}_{6}{\text{H}}_{10}{\text{O}}_{5}⟶5{\text{C}}_{2}{\text{H}}_{5}\text{OH}+5\text{C}{\text{O}}_{2} \end{array}$$

The theoretical maximum yield of broth hexoses and pentoses is 0.511 kg ethanol and 0.489 kg CO_2_ per kg sugar. The overall theoretical ethanol yield (at 20°C) hence becomes 0.719 and 0.736 liters per kg of glucan (and/or other 6C structures) and xylan (and/or other 5C structures), respectively.

*S. cerevisiae*, the yeast commonly used for first generation ethanol production, cannot metabolize xylose. Other yeasts and bacteria are under investigation to ferment xylose and other pentoses into ethanol.

Genetically engineered fungi that produce large volumes of cellulase, xylanase, and hemicellulase enzymes are under investigation. These could convert agricultural residues (e.g., corn stover, straw, and sugar cane bagasse) and energy crops (e.g., switchgrass) into fermentable sugars [33, 35]. Additional research tried to find microorganisms which can effectively ferment both types of sugars into ethanol with* Escherichia coli*,* Klebsiella oxytoca*, and* Zymomonas mobilis* as promising candidates [36, 37].

When using enzymatic hydrolysis, different integration methods of hydrolysis and fermentation steps are proposed. In the separate hydrolysis and fermentation (SHF), the liberated cellulose is treated in a different reactor for hydrolysis and subsequent fermentation than the hydrolysed hemicellulose and lignin. Although this facilitates both the optimization of each separate reactor and the selection of sugar-appropriate microorganisms to ferment the different sugars, the higher investment costs for two separate reactors and the inhibition of the high glucose concentration to fermenting organisms are major disadvantages [38, 39]. Separate hydrolysis and cofermentation (SHCF) and simultaneous saccharification and cofermentation (SSCF) are possible alternatives: cofermenting both C5 and C6 sugars by a single strain of microorganisms in the same reactor significantly improves the process economics and enhances the commercial production of lignocellulosic ethanol in the short term [39–41].

A novel development, the consolidated bioprocessing (CBP) proceeds by producing all required enzymes and ethanol using a single type of microorganisms in a single reactor. CBP is considered as the ultimate evolution of biomass-to-bioethanol conversion technology, since it implies neither capital nor operating costs for dedicated enzyme production together with a reduced consumption of substrate for enzyme production. Unfortunately, it is predicted that it will take several years of research to determine such microorganisms or compatible combinations of microorganisms [41].

With bioethanol production from lignocellulosic biomass, chemical inhibition is a more severe problem than encountered in first generation raw materials. Pretreatment and hydrolysis of lignocellulosics release specific inhibitors, for example, furans, such as furfural and 5-hydroxymethylfurfural (5-HMF), and phenols, such as 4-hydroxybenzaldehyde (4-HB), vanillin, and syringaldehyde, that need to be dealt with to operate hydrolysis and fermentation under optimum conditions and maximum conversion.

To increase the critical ethanol-inhibition concentration, adapted yeasts or bacteria can be used. The most commonly used yeast is* Saccharomyces cerevisiae*, with a moderate yield of fermentation. Research has been done on more promising yeasts and bacteria:* Zymomonas mobilis* succeeds to survive higher ethanol concentrations in the fermenter up to 16 vol%. Not only this advantage, but also a moderate tolerance for acids and sugars, typical inhibitors present in biomass hydrolysis, makes this a very popular yeast for industrial application. The fermentation rate is also higher with* Zymomonas mobilis* in comparison to* Saccharomyces cerevisiae* [1]. An interesting characteristic of* Z. mobilis* is indeed that its plasma membrane contains hopanoids, pentacyclic compounds similar to eukaryotic sterols, thus providing an extraordinary tolerance to ethanol in its environment, around 16 wt%. However, in spite of these attractive advantages, its substrate range is limited to glucose, fructose, and sucrose. It cannot ferment C5 sugars like xylose and arabinose which are important components of lignocellulosic hydrolytes. Unlike yeast,* Z. mobilis* cannot tolerate toxic inhibitors present in lignocellulosic hydrolytes such as acetic acid. Concentration of acetic acid in lignocellulosic hydrolytes can be as high as 1.5 wt%, well above the tolerance threshold of* Z. mobilis*. Several attempts have been made to engineer* Z. mobilis* to overcome its inherent deficiencies by metabolic engineering, mutagenesis, or adaptive mutation to produce acetic acid resistant strains of* Z. mobilis* [42, 43]. However, when these engineered strains metabolize mixed sugars in the presence of inhibitors, the yield and productivity are much lower, thus preventing their industrial application.

To overcome inhibition by hydrolyte components, membrane techniques have been investigated, although further research is certainly required, as described in Section 3.

### 2.5. Purification

Typical ethanol concentrations are in the range of 3–6 vol% only, very low in comparison with 12 to 15 vol% obtained from 1st generation feedstock [1]. Due to the higher water content of the broth, additional distillation efforts are required. Different process improvements, including energy pinch, very high gravity fermentation, and hybrid processes, are described in detail by Kang et al. [1].

### 2.6. Steam and Electricity Generation

The bottom product of the first distillation column (stillage) contains mainly lignin and water next to unconverted cellulose and hemicellulose. This insoluble fraction is dewatered by a pressure filter and sent to a fluidized bed combustor system for steam and electricity generation. This system allows the plant to be self-sufficient in energy supply, reduces solid waste disposal cost, and generates additional revenue through sales of excess electricity [39, 44]. Burning the solid residues for steam and power production is the most beneficial option and meets the energy demand of the plant.

## 3. Current Research Priorities in Biomass to Ethanol

From the previous process assessment, several bottlenecks emerge. Biomass to bioethanol will only be a technical and economic viable alternative to first generation bioethanol, if appropriate solutions are developed. Current production problems hence determine immediate and future research priorities.

Pretreatment, as the first step, accounts for about 33% of the total cost [26]. Better and cost-efficient pretreatment techniques need further investigation, together with methods to reduce or eliminate microbial and chemical contaminants that can reduce the yields. It was already stated that membrane techniques could help to overcome some of the problems, with microfiltration (suspended solids) and ultrafiltration, nanofiltration, or reverse osmosis dealing with dissolved contaminants. The possible application of microfiltration to eliminate suspended solids has recently been confirmed by Kang et al. [3].

In ultrafiltration (UF), solutes of high molecular weight are retained in the so-called retentate, while water and low molecular weight solutes pass through the semipermeable membrane in the permeate. Ultrafiltration is used in industry and research for purifying and concentrating macromolecular (10^3^–10^6^ Da) solutions, especially protein solutions. Removal of suspended solids prior to feeding the membrane is essential to prevent damage to the membrane and minimize the effects of fouling which greatly reduce the separation efficiency.

Nanofiltration and reverse osmosis are high-pressure membrane filtration processes used most often with low total dissolved solids water (surface water and fresh groundwater), for softening (polyvalent cation removal) and removal of disinfection byproduct precursors such as natural organic matter and synthetic organic matter. These membrane separation technologies have been examined in different stages of the bioethanol production.

Enhancing ethanol production by pretreatment involving fungi (e.g.,* T. reesei* and* Basidiomycetes*) with appropriate lignocellulolytic properties at low pH and high temperatures is also a promising and added-value step in SSF ethanol bioconversion. While fungi act slowly, potential lignocellulolytic fungi have been produced by mutagenesis, gene expression, and coculturing [45]. Some genera, such as* Candida*,* Pichia*, and* Dekkera*, were isolated from sugarcane molasses, but resulted in low ethanol concentrations and produced acetic acid, an inhibitor of the fermentative yeast [46]. Some natural wild yeast species appear capable of replacing* S. cerevisiae* in second generation bioethanol [47], but their low bioethanol yield and poor survival in the fermenter need further improvement. As described before, some groups of bacteria such as* Zymomonas mobilis* can convert sugars into ethanol [47], but they are more vulnerable to chemical inhibition than* S. cerevisiae*.

The development of genetically modified fermentative and cellulolytic microorganisms is recommended to increase the ethanol yield and productivity under the stress conditions of high production bioethanol processes [47]. Simultaneous saccharification and fermentation (SSF), simultaneous saccharification and combined fermentation (SSCombF) of the enzymatic hydrolyzate, and CBP are also considered to be cost-effective whilst reducing end-product inhibition. Genetic engineering has succeeded in altering the conventional* S. cerevisiae*'s capacity to ferment glucose and pentose sugars simultaneously [48]. Almeida et al. [49] investigated a modified* S. cerevisiae*, not only capable of cofermenting saccharides but also of generating less furfural inhibitors. As mentioned before, CBP combines hydrolysis and fermentation operations in a single reactor, by using genetically modified microorganisms that produce cellulase enzyme to ferment sugars in a single step. This avoids the costs related to the purchase of cellulolytic enzymes [50]. Lignin should be considered as a valuable energy source, used as a fuel in a CHP and being capable of supplying the power and heat requirements of the complete conversion process.

Genetic engineering, as a powerful biotechnological tool, is required to design new strategies for increasing the ethanol fermentation performance. Upregulation of stress tolerance genes by recombinant DNA technology can be a useful approach to overcome inhibitory situations [51]. Ge et al. [52] obtained three recombinants: HDY-ZMYWBG1, HDY-ZMYWBG2, and HDY-ZMYWBG3 using the lithium acetate transformation method into the* S. cerevisiae* cells. The ethanol yield for HDY-ZMYWBG1 and HDY-ZMYWBG3 is 0.368 g/g and 0.365 g/g, respectively. The resulting consortium was demonstrated to utilize phosphoric acid swollen cellulose (PASC) for growth and ethanol production. The final ethanol production of 1.25 g/L corresponded to 87% of the theoretical value and was 3-fold higher than a similar yeast consortium secreting only the three cellulases [53]. Reconstitution of the* N. crassa* cellodextrin transport system in* Saccharomyces cerevisiae* promotes efficient growth of this yeast on cellodextrins [54, 55]. The engineered yeast strains more rapidly convert cellulose to ethanol when compared with yeast lacking this system. Ha et al. [56] engineered yeasts to coferment mixtures of xylose and cellobiose. It improved ethanol yield when compared to fermentation with either cellobiose or xylose as sole carbon sources. This is a critical step towards enabling economic biofuel production.

Since fermentative microorganisms must be capable of surviving the high temperatures of SSF/SSCombF/CBP processes, further research is required: high temperature ethanol fermentation is an emerging technology provided appropriate microorganisms can be developed. Such high temperature operations do not require cooling and cellulase addition [57]. The thermotolerant yeast,* K. marxianus*, has been documented as a candidate for its ability to coferment both hexose and pentose sugars and survive temperatures of 42–45°C [58].* K. marxianus* was moreover genetically modified to exhibit* T. reesei* and* Aspergillus aculeatus* cellulolytic activities allowing direct and continuous conversion of cellulosic *β*-glucan into ethanol at 48°C, yielding 0.47 g/g ethanol, that is, 92.2% of the theoretical yield, and proving to be an ideal gene modified organism (GMO) for CBP processes [58].

The industrial potential for* S. cerevisiae* fermentation has already been proven for first generation large-scale bioethanol production. Its genetic improvement is gaining increasing research, especially with respect to the CBP option [59, 60], where hydrolysis and substrate fermentation are possible in a single step.

*Z. mobilis* remains an attractive candidate due to its high ethanol yield and resistance to temperatures in the range of 40°C [36]. Numerous genes have been introduced and heterologous expression has been incorporated into* Z. mobilis* to extend its effectiveness toward other substrates, namely, xylose and arabinose [97]. Both the gene engineered* Z. mobilis* and* S. cerevisiae* have proven high ethanol yield and adaptability [98].

Further research is certainly required in optimizing biological pretreatment involving fungi (e.g.,* T. reesei* and Basidiomycetes) that exhibit lignocellulolytic properties at low pH levels and high temperature.

The use of GMOs is questionable, since their introduction into large-scale fermentation operations can pose risks of environmental dissemination and potential exposure risks to public health. Industrial operations using antibiotics to control microbial contaminants in fermenters or as strain markers would generate and release antibiotic resistant organisms and offer another potential environmental and public health risk.

Improvement in each of these individual aspects is required to achieve high conversion and cost-effective biomass-to-bioethanol operations. This needs to be complemented by a comprehensive systems approach, encompassing the different individual steps and accounting for all inputs and outputs during the entire operation regardless of modifications in any of these individual steps.

## 4. Conclusions

The cellulosic bioethanol production process involves specific processing steps, especially in the pretreatment and hydrolysis. Fermentation of C5 and C6 sugars needs adapted microorganisms, still to be further investigated.

New combined processes reduce both the number of operation steps and the production of chemical inhibitors. Recent advances in genetically engineered* S. cerevisiae* and* Z. mobilis* are promising for higher alcohol tolerance and conversion efficiency. Second generation bioethanol could surpass the traditional first generation processes, provided present processing bottlenecks are removed and the best combination of advanced systems is used.

## Figures and Tables

**Table 1:** Common ethanol-petrol mixtures [1, 31].

Code	Composition	Countries	Comments
E5	Max. 5% anhydrous ethanol, min. 95% petrol	Western Europe	Blends for regular cars
E10	Max. 10% anhydrous ethanol, min. 90% petrol	USA, Europe	
E15	Max. 15% anhydrous ethanol, min. 85% petrol	USA, cars >2000	
E25	Max. 25% anhydrous ethanol, min. 75% petrol	Brazil	

E85	Max. 85% anhydrous ethanol, min. 15% petrol	USA, Europe	Flex-fuel vehicles
E100	Hydrous ethanol (~5.3 wt% water)	Brazil	

**Table 2:** Recent literature about second generation bioethanol production.

Reference	Objectives	Main results
[61]	Optimal industrial symbiosis system to improve bioethanol production	(i) Reduced bioethanol production and logistic costs (ii) 2nd generation biomass should be used for bioethanol production

[62]	Bioethanol production from dilute acid pretreated Indian bamboo variety by separate hydrolysis and fermentation	(i) Bioethanol yield of 1.76% (v/v) with an efficiency of 41.69% (ii) Bamboo can be used as feedstock for the production of bioethanol

[63]	Fuel ethanol production from sweet sorghum bagasse using microwave irradiation	(i) An ethanol yield based on total sugar of 480 g kg^−1^ was obtained (ii) Ethanol produced on marginal land at 0.252 m^3^ ton^−1^ biomass

[64]	Ultrasonic-assisted simultaneous SSF of pretreated oil palm fronds for bioethanol production	(i) Maximal bioethanol concentration (18.2 g/L) and yield (57.0%)

[65]	Convert sucrose and homocelluloses in sweet sorghum stalks into ethanol	(i) All sugars in sweet sorghum stalk lignocellulose were hydrolysed into fermentable sugars

[66]	Low-intensity pulsed ultrasound to increase bioethanol production	(i) Increase of the production of bioethanol from lignocellulosic biomass to 52 ± 16%

[67]	Different process configurations for bioethanol production from pretreated olive pruning biomass	(i) Ethanol concentration of 3.7 vol% was obtained

[68]	Bioethanol production from water hyacinth *Eichhornia crassipes *	(i) Yeast *Saccharomyces cerevisiae* TY2 produced ethanol at 9.6 ± 1.1 g/L

[69]	Enhanced saccharification of biologically pretreated wheat straw for ethanol production	(i) Increase of the sugar yield from 33 to 54% and reduction of the quantity of enzymatic mixture by 40%

[70]	Fermentation of biologically pretreated wheat straw for ethanol production	(i) The highest overall ethanol yield was obtained with the yeast *Pachysolen tannophilus*: yielded 163 mg ethanol per gram of raw wheat straw (23 and 35% greater)

[71]	Integration of pulp and paper technology with bioethanol production	(i) Reuse existing assets to the maximum extent (ii) Keep the process as simple as possible (iii) Match the recalcitrance of the biomass with the severity of the pretreatment

[72]	Production of bioethanol by fermentation of lemon peel wastes pretreated with steam explosion	(i) Reduces the residual content of essential oils below 0.025% and decreases the hydrolytic enzyme requirements (ii) Obtained ethanol production in excess of 60 L/1000 kg fresh lemon peel biomass

[73]	Ultrasonic-assisted enzymatic saccharification of sugarcane bagasse for bioethanol production	(i) The maximum glucose yield obtained was 91.28% of the theoretical yield and the maximum amount of glucose obtained was 38.4 g/L (MTCC 7450) (ii) The hydrolyte obtained was 91.22% of the theoretical ethanol yield (MTCC 89) (iii) Decreases the reaction time (iv) The application of low intensity ultrasound enhanced the enzyme release and intensified the enzyme-catalysed reaction

[74]	Status and barriers of advanced biofuel technologies	(i) The major barriers for the commercialization of 2nd generation ethanol production are the high costs of pretreatment, enzymes used in hydrolysis, and conversion of C5 sugars to ethanol (ii) The residues need to be processed for byproducts through biorefinery to improve the economics of the whole process

[75]	Sugarcane bagasse hydrolysis using yeast cellulolytic enzymes	(i) This enzyme extract promoted the conversion of approximately 32% of the cellulose (ii) *C. laurentii* is a good β-glucosidase producer

[76]	Pretreatment of unwashed water-insoluble solids of reed straw and corn stover pretreated with liquid hot water to obtain high concentrations of bioethanol	(i) A high ethanol concentration of 56.28 g/L (reed straw) and 52.26 g/L (corn stover) was obtained (ii) Ethanol yield reached a maximum of 69.1% (reed straw) and 71.1% (corn stover)

[77]	Waste paper sludge as a potential biomass for bioethanol production	(i) SSF using cellulase produced by *A. cellulolyticus* gave ethanol yield 0.208 (g ethanol/g PS organic material) (ii) Consolidated biomass processing (CBP) technology gave ethanol yield 0.19 (g ethanol/g Solka floc)

[78]	Assessment of combinations between pretreatment and conversion configurations for bioethanol production	(i) The process based on dilute acid pretreatment and enzymatic hydrolysis and cofermentation combination shows the best economic potential (ii) The cellulose hydrolysis based on an enzymatic process showed the best energy efficiency

[79]	Combined use of gamma ray and dilute acid for bioethanol production	(i) Increasing enzymatic hydrolysis after combined pretreatment is resulting from or decrease in crystallinity of cellulose, loss of hemicelluloses, and removal or modification of lignin

[80]	Ethanol production from lignocellulosic biomass (exergy analysis)	(i) Lowest environmental impact for second generation bioethanol production (ii) Highest exergy efficiency (steam explosion pretreatment + SSF + dehydration) reaching 79.58%

[81]	Alkaline pretreatment on sugarcane bagasse for bioethanol production	(i) The lowest lignin content (7.16%) was obtained (ii) Cellulose content increased after alkaline pretreatment

[82]	Influence of dual salt pretreatment of sugarcane bagasse for bioethanol production	(i) Better performance was observed using H_2_O_2_ with MnSO_4_·H_2_O and ZnO (ii) The inhibitor formation was limited (iii) The maximum theoretical ethanol yield of 84.32% (13.1 g/L, 0.184 g/g sugarcane bagasse) was achieved during the fermentation

[83]	Bioethanol production from alkaline pretreated sugarcane bagasse using *Phlebia* sp. MG-60	(i) MG-60 produced cellulose and xylanase rapidly during consolidated bioprocessing (CBP) (ii) The maximum theoretical ethanol yield of 65.7% (4.5 g/L) was achieved during the fermentation

[84]	Integrated fungal fermentation of sugarcane bagasse for bioethanol production by *Phlebia* sp. MG-60	(i) 75% moisture content was suitable for subsequent ethanol production (ii) Some additives improved delignification in integrated fungal fermentation (IFF) (iii) Some inorganic chemicals (e.g., Fe^2+^, Mn^2+^, and Cu^2+^) increased the ethanol production

[85]	Furfural and xylose production from sugarcane bagasse in ethanol production	(i) The furfural yield and xylose yield were 6 and 15.5 g/g of sugarcane bagasse, respectively (ii) Ethanol was produced from the residual solid materials obtained from furfural and xylose at 87.4% and 89.3%, respectively

**Table 3:** Potential lignocellulosic biomass sources and compositions (% dry weight) [86, 87].

Raw material	Hemicelluloses	Cellulose	Lignin	Others (i.e., ash)
Agricultural residues	25–50	37–50	5–15	12–16
Hardwood	25–40	45–47	20–25	0.80
Softwood	25–29	40–45	30–60	0.50
Grasses	35–50	25–40	—^a^	2–5
Waste papers from chemical pulps	12–20	50–70	6–10	2
Newspaper	25–40	40–55	18–30	5–8
Switch grass	30–35	40–45	12	4-5

^a^Not present or not available.

**Table 4:** Ultimate and proximate analyses of different biomasses (wt%).

Ultimate analysis (wt% on dry basis)						Proximate analysis			
Sample	C	O	H	N	S	VM	M	FC	A
Wood and woody biomass									
Pine	54.5	38.7	5.9	0.5	0.42	46.1	37.8	12.9	3.2
Eucalyptus bark	48.7	45.3	5.7	0.3	0.05	68.7	12	15.1	4.2
Forest residue	52.7	41.1	5.4	0.7	0.10	34.5	56.8	7.3	1.4
Land clearing wood	50.7	42.8	6	0.4	0.07	35.4	49.2	7	8.4
Olive wood	49	44.9	5.4	0.7	0.03	74.3	6.6	16.1	3
Pine chips	52.8	40.5	6.1	0.5	0.09	66.9	7.6	20	5.5
Pine sawdust	51	42.9	6	0.1	0.01	70.4	15.3	14.2	0.1
Poplar	51.6	41.7	6.1	0.6	0.02	79.7	6.8	11.5	2
Mixed sawdust	49.8	43.7	6	0.5	0.02	55.1	34.9	9.3	0.7
Spruce wood	52.3	41.2	6.1	0.3	0.10	75.7	6.7	17.1	0.5
Willow	49.8	43.4	6.1	0.6	0.06	74.2	10.1	14.3	1.4

Herbaceous and agriculture biomass									
Bamboo	52	42.5	5.1	0.4	0.04	71	13	15.2	0.8
Miscanthus grass	49.2	44.2	6	0.4	0.15	71.9	11.4	14	2.7
Sweet sorghum	49.7	43.7	6.1	0.4	0.09	71.8	7	16.8	4.4
Switchgrass	49.7	43.4	6.1	0.7	0.11	70.8	11.9	12.8	4.5
Corn straw	48.7	44.1	6.4	0.7	0.08	67.4	7.4	17.8	7.1
Rice straw	50.1	43	5.7	1	0.16	59.4	7.6	14.4	18.6
Wheat straw	49.4	43.6	6.1	0.7	0.17	67.2	10.1	16.3	6.4
Coconut shell	51.1	43.1	5.6	0.1	0.1	70.5	4.4	22	3.1
Cotton husks	50.4	39.8	8.4	1.4	0.01	73	6.9	16.9	3.2
Corn stover	42.5	42.6	5	0.8	NA	78.1	10.6	17.6	3.7
Groundnut shell	50.9	40.4	7.5	1.2	0.02	68.1	7.9	20.9	3.1
Hazelnut shell	51.5	41.6	5.5	1.4	0.04	71.5	7.2	19.9	1.4
Olive husks	50	42.1	6.2	1.6	0.04	73.7	6.8	17.4	2.1
Rice husks	49.3	43.7	6.1	0.8	0.22	56.1	10.6	17.2	16.1
Soya husks	45.4	46.9	6.7	0.9	0.08	69.6	6.3	19	5.1
Bagasse	49.8	43.9	6	0.2	0.08	76.6	10.4	11.1	1.9
Sunflower husks	50.4	43	5.5	1.1	0.1	69.1	9.1	19	2.8
Tea wastes	48.6	42.2	5.4	3.8		70.3	7.26	18.57	3.88

Other biomass sources									
Chicken litter	60.5	25.3	6.8	6.2	1.2	43.3	9.3	13.1	34.3
Agricultural residue	52.4	41.2	6	0.4	0.04	54.7	30.3	12.7	2.3
Mixed waste paper	52.3	40.2	7.2	0.2	0.08	76.8	8.8	6.8	7.6
Refuse-derived fuel	53.8	36.8	7.8	1.1	0.47	70.3	4.2	0.5	25
Sewage sludge	50.9	33.4	7.3	6.1	2.33	45	6.4	5.3	43.3
Wood yard waste	52.2	40.4	6	1.1	0.3	40.9	38.1	8.4	12.6

VM: volatile matter; M: moisture; FC: fixed carbon; A: ash.

**Table 5:** Elemental ash composition of different biomass.

Sample	SiO_2_	CaO	K_2_O	P_2_O_5_	Al_2_O_3_	MgO	Fe_2_O_3_	SO_3_	Na_2_O	TiO_2_
Wood and woody biomass										
Eucalyptus bark	10.04	57.74	9.29	2.35	3.1	10.91	1.12	3.47	1.86	0.12
Poplar bark	1.86	77.31	8.93	2.48	0.62	2.36	0.74	0.74	4.84	0.12
Willow	6.1	46.09	23.4	13.01	1.96	4.03	0.74	3	1.61	0.06
Wood residue	53.15	11.66	4.85	1.37	12.64	3.06	6.24	1.99	4.47	0.57

Herbaceous and agriculture biomass										
Bamboo whole	9.92	4.46	53.38	20.33	0.67	6.57	0.67	3.68	0.31	0.01
Miscanthus	56.42	10.77	19.75	5.54	0.79	3.01	0.94	2.28	0.47	0.03
Sorghum grass	73.21	7.02	8.97	4.43	1.83	2.21	0.95	1.11	0.25	0.02
Sweet sorghum	66.85	10.41	4.49	3.47	0.81	3.12	0.58	3.47	1.47	0.06
Switchgrass	66.25	10.21	9.64	3.92	2.22	4.71	1.36	0.83	0.58	0.28
Wheat straw	50.35	8.21	24.89	3.54	1.54	2.74	0.88	4.24	3.52	0.09
Rice husks	94.48	0.97	2.29	0.54	0.21	0.19	0.22	0.92	0.16	0.02
Sugar cane bagasse	46.79	4.91	6.95	3.87	14.6	4.56	11.12	3.57	1.61	2.02
Sunflower husks	23.66	15.31	28.53	7.13	8.75	7.33	4.27	4.07	0.8	0.15

Other biomass varieties										
Chicken litter	5.77	56.85	12.19	15.4	1.01	4.11	0.45	3.59	0.6	0.03
Mixed waste paper	28.62	7.63	0.16	0.2	53.53	2.4	0.82	1.73	0.54	4.37
Refuse-derived fuel	38.67	26.81	0.23	0.77	14.54	6.45	6.26	3.01	1.36	1.9
Sewage sludge	33.28	13.04	1.6	15.88	12.91	2.49	15.7	2.05	2.25	0.8
Wood yard waste	60.1	23.92	2.98	1.98	3.08	2.17	1.98	2.46	1.01	0.32

**Table 6:** Assessment of selected pretreatment processes [15, 88–96].

Pretreatment process	Yield of fermentable sugars	Wastes	Investment
Physical or physicochemical			
(i) Mechanical	Low	Very low	Low
(ii) Steam explosion	High	Low	High
(iii) Ammonia fiber explosion (AFEX)	Moderate	Very low	High
(iv) Carbonic acid	Very high	Very low	Low

Chemical			
(i) Dilute acid	Very high	High	Moderate
(ii) Concentrated acid	Very high	High	High
(iii) Alkaline extraction	Very high	High	Low
(iv) Wet oxidation	High	Low	Low
(v) Organosolv	Very high	Low	Very high

## References

[1] Kang Q., Appels L., Baeyens J., Dewil R., Tan T. (2014). Energy-efficient production of cassava-based bio-ethanol. *Advances in Bioscience and Biotechnology*.

[2] Shen L., Lei J., Bi Y. (2011). Performance and emission characteristics of diesel engine fueled with ethanol-diesel blends in different altitude regions. *Journal of Biomedicine and Biotechnology*.

[3] Kang Q., Huybrechts J., van der Bruggen B., Baeyens J., Tan T. W., Dewil R. (2014). Hydrophilic membranes to replace molecular sieves in dewatering the bio-ethanol/water azeotropic mixture. *Separation and Purification Technology*.

[4] Fischer G., Schrattenholzer L. (2001). Global bioenergy potentials through 2050. *Biomass & Bioenergy*.

[5] Demirbas M. F., Balat M., Balat H. (2009). Potential contribution of biomass to the sustainable energy development. *Energy Conversion and Management*.

[6] Werther J., Saenger M., Hartge E.-U., Ogada T., Siagi Z. (2000). Combustion of agricultural residues. *Progress in Energy and Combustion Science*.

[7] Bääth H., Gällerspäng A., Hallsby G. (2002). Remote sensing, field survey, and long-term forecasting: an efficient combination for local assessments of forest fuels. *Biomass and Bioenergy*.

[8] Malinen J., Pesonen M., Määttä T., Kajanus M. (2001). Potential harvest for wood fuels (energy wood) from logging residues and first thinnings in Southern Finland. *Biomass and Bioenergy*.

[9] Demirbaş A. (2001). Energy balance, energy sources, energy policy, future developments and energy investments in Turkey. *Energy Conversion and Management*.

[10] Raven P. H., Evert R. F., Eichhorn S. E. (1999). *Biology of Plants*.

[11] Ebringerova A., Hromadkova Z., Heinze T. (2005). Hemicellulose. *Advances in Polymer Science*.

[12] Deguchi S., Mukai S.-A., Tsudome M., Horikoshi K. (2006). Facile generation of fullerene nanoparticles by hand-grinding. *Advanced Materials*.

[13] Saha B. C. (2003). Hemicellulose bioconversion. *Journal of Industrial Microbiology and Biotechnology*.

[14] Girio F. M., Fonseca C., Carvalheiro F., Duarte L. C., Marques S., Bogel-Lukasic R. (2010). Hemicellulose. *Bioresource Technology*.

[15] Hendriks A. T. W. M., Zeeman G. (2009). Pretreatments to enhance the digestibility of lignocellulosic biomass. *Bioresource Technology*.

[16] Ladisch M. R., Mosier N. S., Kim Y., Ximenes E., Hogsett D. (2010). Converting cellulose to biofuels. SBE special supplement biofuels. *Chemical Engineering Progress*.

[17] Chen F., Srinivasa R. M. S., Temple S., Jackson L., Shadle G., Dixon R. A. (2006). Multi-site genetic modulation of monolignol biosynthesis suggests new routes for formation of syringyl lignin and wallbound ferulic acid in alfalfa (*Medicago sativa* L.). *Plant Journal*.

[18] Li X., Weng J.-K., Chapple C. (2008). Improvement of biomass through lignin modification. *Plant Journal*.

[19] Demirbas A. (2004). Combustion characteristics of different biomass fuels. *Progress in Energy and Combustion Science*.

[20] Mahmoudi S., Baeyens J., Seville J. P. K. (2010). NO_x_ formation and selective non-catalytic reduction (SNCR) in a fluidized bed combustor of biomass. *Biomass and Bioenergy*.

[21] Shen J., Zhu S., Liu X., Zhang H., Tan J. (2010). The prediction of elemental composition of biomass based on proximate analysis. *Energy Conversion and Management*.

[22] van Loo S., Koppenjan J. (2008). *The Handbook of Biomass Combustion and Cofiring*.

[23] Telmo C., Lousada J., Moreira N. (2010). Review: proximate analysis, backwards stepwise regression between gross calorific value, ultimate and chemical analysis of wood. *Bioresource Technology*.

[24] Sun Y., Cheng J. (2002). Hydrolysis of lignocellulosic materials for ethanol production: a review. *Bioresource Technology*.

[25] U.S. Department of Energy (DOE) (2000). The DOE bioethanol pilot plant.

[26] Tomás-Pejó E., Oliva J. M., Ballesteros M. (2008). Realistic approach for full-scale bioethanol production from lignocellulose: a review. *Journal of Scientific and Industrial Research*.

[27] Zhang Y. J., Li Q., Su J. M. (2015). A green and efficient technology for the degradation of cellulosic materials: structure changes and enhanced enzymatic hydrolysis of natural cellulose pretreated by synergistic interaction of mechanical activation and metal salt. *Bioresource Technology*.

[28] Singh A., Bajar S., Bishnoi N. R. (2014). Enzymatic hydrolysis of microwave alkali pretreated rice husk for ethanol production by *Saccharomyces cerevisiae*, *Scheffersomyces stipitis* and their co-culture. *Fuel*.

[29] Maitan-Alfenas G. P., Visser E. M., Guimarães V. M. (2015). Enzymatic hydrolysis of lignocellulosic biomass: converting food waste in valuable products. *Current Opinion in Food Science*.

[30] Cui X. H., Zhao X. B., Zeng J., Loh S. K., Choo Y. M., Liu D. H. (2014). Robust enzymatic hydrolysis of formiline-pretreated oil palm empty fruit bunches (EFB) for efficient conversion of polysaccharide to sugars and ethanol. *Bioresource Technology*.

[31] Baeyens J., Kang Q., Appels L., Dewil R., Lv Y., Tan T. (2015). Challenges and opportunities in improving the production of bio-ethanol. *Progress in Energy and Combustion Science*.

[32] Chandel A. K., da Silva S. S., Singh O. V. (2013). Detoxification of lignocellulose hydrolysates: biochemical and metabolic engineering toward white biotechnology. *Bioenergy Research*.

[33] Mousdale D. M. (2008). Biofuels: biotechnology, chemistry and sustainable development. *The Iogen Corporation Process as a Template and Paradigm*.

[34] Lindstedt J. Alcohol production from lignicellulosic feedstock.

[35] Deswal D., Gupta R., Nandal P., Kuhad R. C. (2014). Fungal pretreatment improves amenability of lignocellulosic material for its saccharification to sugars. *Carbohydrate Polymers*.

[36] Dien B. S., Cotta M. A., Jeffries T. W. (2003). Bacteria engineered for fuel ethanol production: current status. *Applied Microbiology and Biotechnology*.

[37] Hahn-Hägerdal B., Karhumaa H. B. K., Fonseca C., Spencer-Martins I., Gorwa-Grauslund M. F. (2007). Towards industrial pentose-fermenting yeast strains. *Applied Microbiology and Biotechnology*.

[38] Aden A., Foust T. (2009). Technoeconomic analysis of the dilute sulfuric acid and enzymatic hydrolysis process for the conversion of corn stover to ethanol. *Cellulose*.

[39] Kazi F. K., Fortman J. A., Anex R. P. (2010). Techno-economic comparison of process technologies for biochemical ethanol production from corn stover. *Fuel*.

[40] Humbird D., Aden A. (2009). Biochemical production of ethanol from Corn Stover: 2008 State of Technology model. *NREL Report No.*.

[41] Klein-Marcuschamer D., Oleskowicz-Popiel P., Simmons B. A., Blanch H. W. (2010). Technoeconomic analysis of biofuels: a wiki-based platform for lignocellulosic biorefineries. *Biomass and Bioenergy*.

[42] Joachimsthal E. L., Rogers P. L. (2000). Characterization of a high-productivity recombinant strain of *Zymomonas mobilis* for ethanol production from glucose/xylose mixtures. *Applied Biochemistry and Biotechnology*.

[43] Chen R., Wang Y., Shin H. D., Agrawal M., Mao Z. C. Strains of *Zymomonas mobilis* for fermentation of biomass.

[44] Humbird D., Davis R., Tao L. (2011). Process design and economics for biochemical conversion of lignocellulosic biomass to ethanol.

[45] Dashtban M., Schraft H., Qin W. (2009). Fungal bioconversion of lignocellulosic residues; opportunities & perspectives. *International Journal of Biological Sciences*.

[46] Basílio A. C. M., de Araújo P. R. L., de Morais J. O. F., da Silva Filho E. A., de Morais M. A., Simões D. A. (2008). Detection and identification of wild yeast contaminants of the industrial fuel ethanol fermentation process. *Current Microbiology*.

[47] Chen Y. C. B. (2009). *Initial investigation of xylose fermentation for lignocellulosic bioethanol production [Ph.D. thesis]*.

[48] Ho N. W. Y., Chen Z. D., Brainard A. P. (1998). Genetically engineered *Saccharomyces* yeast capable of effective cofermentation of glucose and xylose. *Applied and Environmental Microbiology*.

[49] Almeida J. R. M., Modig T., Röder A., Lidén G., Gorwa-Grauslund M.-F. (2008). *Pichia stipitis* xylose reductase helps detoxifying lignocellulosic hydrolysate by reducing 5-hydroxymethyl-furfural (HMF). *Biotechnology for Biofuels*.

[50] Lynd L. R., van Zyl W. H., McBride J. E., Laser M. (2005). Consolidated bioprocessing of cellulosic biomass: an update. *Current Opinion in Biotechnology*.

[51] Doğan A., Demirci S., Aytekin A. Ö., Şahin F. (2014). Improvements of tolerance to stress conditions by genetic engineering in *Saccharomyces cerevisiae* during ethanol production. *Applied Biochemistry and Biotechnology*.

[52] Ge J. P., Zhang L. Y., Ping W. X., Zhang M. Y., Shen Y., Song G. (2014). Genetically engineered *Saccharomyces cerevisiae* strain that can ultilize both xylose and glucose for fermentation. *Applied Mechanics and Materials*.

[53] Goyal G., Tsai S.-L., Madan B., DaSilva N. A., Chen W. (2011). Simultaneous cell growth and ethanol production from cellulose by an engineered yeast consortium displaying a functional mini-cellulosome. *Microbial Cell Factories*.

[54] Galazka J. M., Tian C., Beeson W. T., Martinez B., Glass N. L., Cate J. H. D. (2010). Cellodextrin transport in yeast for improved biofuel production. *Science*.

[55] Ha S.-J., Wei Q., Kim S. R., Galazka J. M., Cate J., Jin Y.-S. (2011). Cofermentation of cellobiose and galactose by an engineered *Saccharomyces cerevisiae* Strain. *Applied and Environmental Microbiology*.

[56] Ha S.-J., Galazka J. M., Kim S. R. (2011). Engineered *Saccharomyces cerevisiae* capable of simultaneous cellobiose and xylose fermentation. *Proceedings of the National Academy of Sciences of the United States of America*.

[57] Fonseca G. G., Heinzle E., Wittmann C., Gombert A. K. (2008). The yeast *Kluyveromyces marxianus* and its biotechnological potential. *Applied Microbiology and Biotechnology*.

[58] Yanase S., Hasunuma T., Yamada R. (2010). Direct ethanol production from cellulosic materials at high temperature using the thermotolerant yeast *Kluyveromyces marxianus* displaying cellulolytic enzymes. *Applied Microbiology and Biotechnology*.

[59] van Zyl W. H., Lynd L. R., den Haan R., McBride J. E. (2007). Consolidated bioprocessing for bioethanol production using *Saccharomyces cerevisiae*. *Advances in Biochemical Engineering/Biotechnology*.

[60] Lilly M., Fierobe H.-P., van Zyl W. H., Volschenk H. (2009). Heterologous expression of a *Clostridium* minicellulosome in *Saccharomyces cerevisiae*. *FEMS Yeast Research*.

[61] Gonela V., Zhang J. (2014). Design of the optimal industrial symbiosis system to improve bioethanol production. *Journal of Cleaner Production*.

[62] Sindhu R., Kuttiraja M., Binod P., Sukumaran R. K., Pandey A. (2014). Bioethanol production from dilute acid pretreated Indian bamboo variety (*Dendrocalamus* sp.) by separate hydrolysis and fermentation. *Industrial Crops and Products*.

[63] Marx S., Ndaba B., Chiyanzu I., Schabort C. (2014). Fuel ethanol production from sweet sorghum bagasse using microwave irradiation. *Biomass and Bioenergy*.

[64] Ofori-Boateng C., Lee K. T. (2014). Ultrasonic-assisted simultaneous saccharification and fermentation of pretreated oil palm fronds for sustainable bioethanol production. *Fuel*.

[65] Li J. H., Li S. Z., Han B., Yu M. H., Li G. M., Jiang Y. (2013). A novel cost-effective technology to convert sucrose and homocelluloses in sweet sorghum stalks into ethanol. *Biotechnology for Biofuels*.

[66] Shaheen M., Choi M., Ang W. (2013). Application of low-intensity pulsed ultrasound to increase bio-ethanol production. *Renewable Energy*.

[67] Manzanares P., Negro M. J., Oliva J. M. (2011). Different process configurations for bioethanol production from pretreated olive pruning biomass. *Journal of Chemical Technology & Biotechnology*.

[68] Takagi T., Uchida M., Matsushima R., Ishida M., Urano N. (2012). Efficient bioethanol production from water hyacinth *Eichhornia crassipes* by both preparation of the saccharified solution and selection of fermenting yeasts. *Fisheries Science*.

[69] López-Abelairas M., Lu-Chau T. A., Lema J. M. (2013). Enhanced saccharification of biologically pretreated wheat straw for ethanol production. *Applied Biochemistry and Biotechnology*.

[70] López-Abelairas M., Lu-Chau T. A., Lema J. M. (2013). Fermentation of biologically pretreated wheat straw for ethanol production: comparison of fermentative microorganisms and process configurations. *Applied Biochemistry and Biotechnology*.

[71] Phillips R. B., Jameel H., Chang H. M. (2013). Integration of pulp and paper technology with bioethanol production. *Biotechnology for Biofuels*.

[72] Boluda-Aguilar M., López-Gómez A. (2013). Production of bioethanol by fermentation of lemon (*Citrus limon* L.) peel wastes pretreated with steam explosion. *Industrial Crops and Products*.

[73] Velmurugan R., Muthukumar K. (2012). Sono-assisted enzymatic saccharification of sugarcane bagasse for bioethanol production. *Biochemical Engineering Journal*.

[74] Cheng J. J., Timilsina G. R. (2011). Status and barriers of advanced biofuel technologies: a review. *Renewable Energy*.

[75] de Souza A. C., Carvalho F. P., e Batista C. F. S., Schwan R. F., Dias D. R. (2013). Sugarcane bagasse hydrolysis using yeast cellulolytic enzymes. *Journal of Microbiology and Biotechnology*.

[76] Lu J., Li X., Yang R. F., Zhao J., Qu Y. B. (2013). Tween 40 pretreatment of unwashed water-insoluble solids of reed straw and corn stover pretreated with liquid hot water to obtain high concentrations of bioethanol. *Biotechnology for Biofuels*.

[77] Prasetyo J., Park E. Y. (2013). Waste paper sludge as a potential biomass for bio-ethanol production. *Korean Journal of Chemical Engineering*.

[78] Conde-Mejía C., Jiménez-Gutiérrez A., El-Halwagi M. M. (2013). Assessment of combinations between pretreatment and conversion configurations for bioethanol production. *ACS Sustainable Chemistry and Engineering*.

[79] Hong S. H., Lee J. T., Lee S. (2014). Improved enzymatic hydrolysis of wheat straw by combined use of gamma ray and dilute acid for bioethanol production. *Radiation Physics and Chemistry*.

[80] Ojeda K., Sánchez E., Kafarov V. (2011). Sustainable ethanol production from lignocellulosic biomass-application of exergy analysis. *Energy*.

[81] Maryana R., Ma'rifatun D., Wheni A. I., Satriyo K. W., Angga Rizal W. (2014). Alkaline pretreatment on sugarcane bagasse for bioethanol production. *Energy Procedia*.

[82] Ramadoss G., Muthukumar K. (2015). Influence of dual salt on the pretreatment of sugarcane bagasse with hydrogen peroxide for bioethanol production. *Chemical Engineering Journal*.

[83] Khuong L. D., Kondo R., de Leon R., Anh T. K., Shimizu K., Kamei I. (2014). Bioethanol production from alkaline-pretreated sugarcane bagasse by consolidated bioprocessing using *Phlebia* sp. MG-60. *International Biodeterioration and Biodegradation*.

[84] Duy Khuong L., Kondo R., de Leon R. (2014). Effect of chemical factors on integrated fungal fermentation of sugarcane bagasse for ethanol production by a white-rot fungus, *Phlebia* sp. MG-60. *Bioresource Technology*.

[85] Mesa L., Morales M., González E. (2014). Restructuring the processes for furfural and xylose production from sugarcane bagasse in a biorefinery concept for ethanol production. *Chemical Engineering and Processing: Process Intensification*.

[86] Swart J. A. A., Jiang J., Ho P. (2008). Risk perceptions and GM crops: the case of China. *Tailoring Biotechnologies: The Socialization of Science and Technology*.

[87] Harel A.

[88] Gurgel L. V. A., Pimenta M. T. B., Curvelo A. A. S. (2014). Enhancing liquid hot water (LHW) pretreatment of sugarcane bagasse by high pressure carbon dioxide (HP-CO_2_). *Industrial Crops and Products*.

[89] Behera S., Arora R., Nandhagopal N., Kumar S. (2014). Importance of chemical pretreatment for bioconversion of lignocellulosic biomass. *Renewable & Sustainable Energy Reviews*.

[90] Zeng J. J., Tong Z. H., Wang L. T., Zhu J. Y., Ingram L. (2014). Isolation and structural characterization of sugarcane bagasse lignin after dilute phosphoric acid plus steam explosion pretreatment and its effect on cellulose hydrolysis. *Bioresource Technology*.

[91] Phan D. T., Tan C. S. (2014). Innovative pretreatment of sugarcane bagasse using supercritical CO_2_ followed by alkaline hydrogen peroxide. *Bioresource Technology*.

[92] Prado J. M., Follegatti-Romero L. A., Forster-Carneiro T., Rostagno M. A., Maugeri Filho F., Meireles M. A. A. (2014). Hydrolysis of sugarcane bagasse in subcritical water. *Journal of Supercritical Fluids*.

[93] Singh J., Suhag M., Dhaka A. (2015). Augmented digestion of lignocellulose by steam explosion, acid and alkaline pretreatment methods: a review. *Carbohydrate Polymers*.

[94] Singh R., Shukla A., Tiwari S., Srivastava M. (2014). A review on delignification of lignocellulosic biomass for enhancement of ethanol production potential. *Renewable and Sustainable Energy Reviews*.

[95] Biswas R., Uellendahl H., Ahring B. K. (2014). Wet explosion pretreatment of sugarcane bagasse for enhanced enzymatic hydrolysis. *Biomass & Bioenergy*.

[96] Sharma S., Kumar R., Gaur R. (2014). Pilot scale study on steam explosion and mass balance for higher sugar recovery from rice straw. *Bioresource Technology*.

[97] Deanda K., Zhang M., Eddy C., Picataggio S. (1996). Development of an arabinose-fermenting *Zymomonas mobilis* strain by metabolic pathway engineering. *Applied and Environmental Microbiology*.

[98] Weber C., Farwick A., Benisch F. (2010). Trends and challenges in the microbial production of lignocellulosic bioalcohol fuels. *Applied Microbiology and Biotechnology*.

